# Cleft Palate Repair with Orbicularis Oris Plus Buccal Mucosal Flap: A New Double Layered-Technique

**DOI:** 10.30476/dentjods.2022.94504.1795

**Published:** 2023-09

**Authors:** Nihat Akbulut, Ahmet Altan, Esengul Sen

**Affiliations:** 1 Dept. of Oral and Maxillofacial Surgery, Ondokuz Mayıs University, Faculty of Dentistry, Samsun, Turkey; 2 Dept. of Oral and Maxillofacial Surgery, Necmettin Erbakan University, Faculty of Dentistry, Konya, Turkey; 3 Dept. of Oral and Maxillofacial Surgery, Gaziosmanpasa University, Faculty of Dentistry, Tokat, Turkey

**Keywords:** Cleft lip, Cleft palate, Orofacial cleft, Surgical flap

## Abstract

Recurrent oronasal fistula closure is a challenging phenomenon that has been managed with many surgical or flap techniques, such as local, regional, and distant flaps, with various modifications. Despite these options, the ideal method to repair this kind of chronic fistula has not yet been established. It is difficult to repair because recurrent surgical repairs or interventions cause this region to become more fibrotic with less vascular tissue, which considerably reduces the likelihood of closing this kind of fistula. For this reason, surgeons and researchers continue to work to overcome these obstacles by using more regional, vascular, and neighboring tissue. Classic cleft palate repair techniques use double-layered, nasal, and oral side closure and even a three-layered technique (e.g. plus levator veli palatini and tensor veli palatini muscular repair) in the soft palate region. Hence, we used partial orbicularis oris muscle with enough vascular supply to repair the nasal side and cheek mucosal flap to repair the oral side as a double-layered repair technique. Two years later, during routine patient follow-up, no complications were identified, and the patient’s satisfaction with this treatment was acceptable.

## Introduction

Cleft lip and palate is a common entity found in oral and maxillofacial surgery that is usually repaired during childhood. However, it sometimes re-opens, especially in the anterior part of the palate, and can cause dentofacial deformity (e.g. skeletal Class III malocclusion) [ 1 - 2 ]. 

The cause of dehiscence of the oronasal track may be iatrogenic; it can be due to a complication of surgical repair, which has a relatively high incidence rate of 4-34%, or radiotherapy [ 1 - 5 ]. In addition, recurrent surgeries for a chronic oronasal fistula reduce the likelihood of successful repair due to excessive tissue fibrosis. Moreover, diminished blood flow, increasing age, and decreasing regenerative capacity during this period present challenges [ 1 - 3 ]. 

Concerning the complications of recurrent surgeries, oronasal fistulas have been repaired with many surgical techniques. Honnebier *et al*. [ 6 ] classified the techniques employed for fistula closure into two groups: mucoperiosteal flaps in a single shape and hinge flaps including additional tissue from the region nearby the fistula as buccal mucosa or tongue tissue [ 3 ].

 In addition to these techniques, faced with dehiscence of the flap, the closure technique emerges as a buccal mucosal flap, orbicularis oris flap, tongue flap, and free flaps as dermis or mucosal and myomucosal flaps, bone, and conchal graft. If a successful result cannot be achieved, prosthetic obturation of the fistula can be made [ 3 ]. Tiwari and Sarabahi [ 2 ] used the orbicularis oris muscle with mucosa for the closure of an oronasal fistula as a single-layer technique.

In this technical paper, we present a case of a chronic oronasal fistula that had been repaired many times previously that we treated with a new double-layered technique.

## Case Presentation

**Figure 1:**
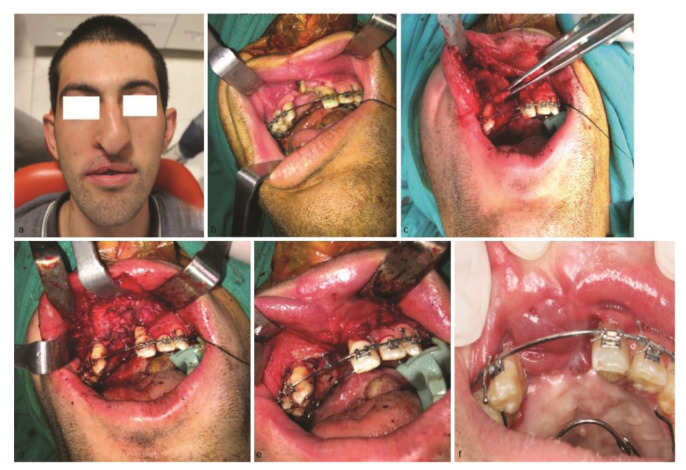
Pretreatment view of the patient, **a:** Preoperative extraoral view of the patient, **b:** Preoperative intraoral view of the patient showing the oronasal fistula, **c:** Appearance of the partial orbicularis oris flap, **d:** Appearance of the suturing of the flap to the nasal side of the fistula, **e:** Appearance of the cheek flap suturing to the oral side of the fistula, **f:** Two years later view of the surgical area showing the uneventful healing

**Figure 2:**
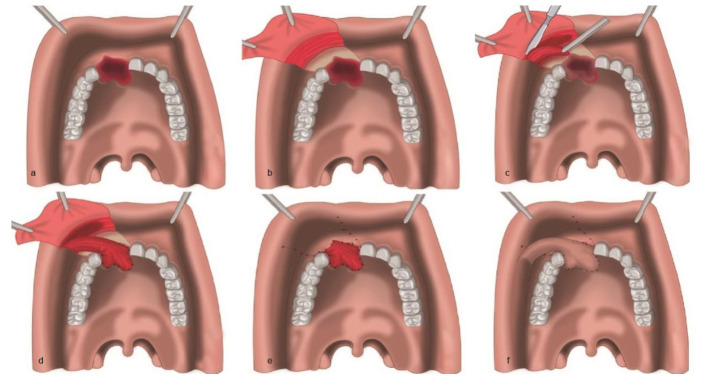
Representative drawing of the surgical technique, **a:** Appearance of the just incised of the fistula edges, **b:** Appearance of the uncovering of the orbicularis oris muscle, **c:** Dissecting of the partial orbicularis oris flap, **d:** Suturing of the partial orbicularis oris flap to the nasal side of the fistula, **e:** The suturing of the mucosa over of the remaining of the orbicularis oris muscle, **f:** Appearance of the cheek flap suturing to the oral side of the fistula

**Figure 3:**
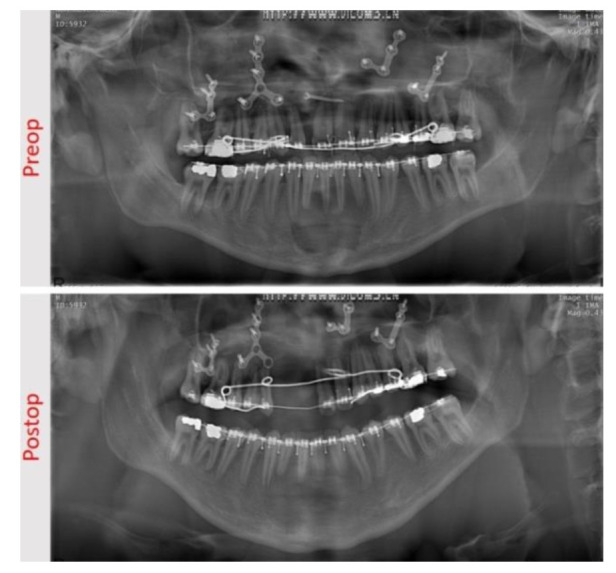
Pre-treatment and post-treatment extra-oral view of the patient, in which the functional lip movement could be seen

**Figure 4:**
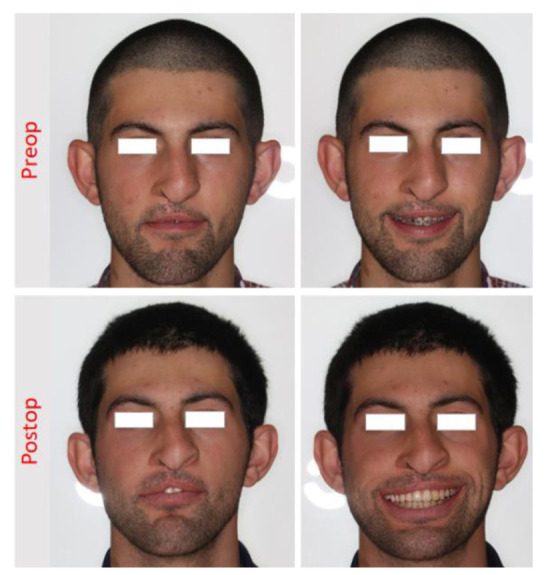
The removable prosthesis was constructed and applied to the patient

An 18-year-old male patient was referred to Gaziosmanpaşa University, Faculty of Dentistry, Orthodontics, and Oral and Maxillofacial Surgery Clinic for skeletal Class III malocclusion and anterior palato-alveolar fistula. He had undergone multiple operations throughout childhood and adolescence. Clinical and radiological examinations were carried out by both Orthodontics and Oral and Maxillofacial Surgery Clinic, and these clinics decided to conduct multidisciplinary treatment. He had no relevant systemic illnesses. An informed consent form regarding the patient’s treatment steps and the free use of patient data for scientific or academic purposes was completed. Orthodontics and Oral and Maxillofacial Surgery Clinic decided on maxillo-mandibular double jaw surgery plus surgical closure of the fistula using a chin bone graft and primary suturation of the adjacent mucoperiosteal flap after initial classic orthodontic treatment was completed. These plans were carried out, but the fistula finally was re-opened. Therefore, after removing the failed bone graft from the surgical site, we used a new technique with double- layered flaps (Figure 1-3). This included using partial orbicularis oris muscle for nasal floor closure (Figure 1) that originated from the modiolus and extended to the surgical area. A cheek mucosal flap was used for oral side closure due to not debulking much of the inefficient volume of the lip that originated from the right cheek, and it was extended to the surgical area. At his follow-up appointment, two years later, successful healing of the chronic oronasal fistula was observed. During this period and in the follow-up period, the removable prosthesis was constructed and applied to the patient (Figure 4). The patient is undergoing regular follow-ups.

## Discussion

Chronic oronasal fistula is not a rare phenomenon after cleft palate and alveolus repair [ 1 , 3 , 5 - 6 ]. Because of fibrotic tissue formation and the failure the regenerative capacity of these tissues, along with advancing age, recurrent surgeries usually result in less vascularity in the surgical region. For these reasons, a cleft palate that is resistant to surgical closure may be challenging for surgeons, and the ideal closure technique has not yet been established [ 3 , 6 ]. There are several traditional methods used by various surgeons that apply adjacent, regional, and distant tissues to repair these difficult cases, including anterior palatal fistula [ 2 ].

To overcome the problematic issue of diminished blood supply to this fibrotic tissue, Abdel-Aziz *et al*. [ 3 ] used a myomucosal superior lip flap with a successful outcome for closure of an anterior palatal fistula, like our case, in which we used partial orbicularis oris muscle to supply adequate blood nutrition to the surgical site tissues. Before the successful outcome of our new double-layered closure technique, traditional techniques, such as surrounding mucoperiosteal flaps closure, were once used for our case with Le Fort 1 and grafting and subsequently other labial flaps surgery, but they failed.

Tiwari and Sarabahi [ 2 ] used the orbicularis oris muscle for the closure of an anterior palate defect with a single layer flap that resembles our technique. In our flaps, however, a partial orbicularis oris muscle flap was used for closure of the nasal floor, and a cheek flap also was used for oral side closure in a separate manner, used as a double-layered technique. Honnebier *et al*. [ 6 ] used the free buccal flap to close the nasal side and protect the oral side closure of the less vascular mucoperiosteal flap. Contrary to Honnebier *et al*. [ 6 ], we used a partial orbicularis oris muscle flap with rich blood supply to close the nasal side and a regional buccal mucosal flap with less blood supply to strengthen the nasal side closure.

Some authors used Veau flaps with buccal mucosal flaps for fistula closure along with bone graft to fill the bony defect [ 2 ]. We initially used a chin bone graft to fill the bony gap of the fistula with buccal and labial flaps for the closure of the Le Fort 1 surgical sites, but, as mentioned above, it re-opened. Therefore, we used this new technique to repair the fistula after removing the failed bone graft from the surgical area.

The following are the main advantages of our new double-layered (partial orbicularis oris plus cheek mucosal flap) technique:

1- This technique is safe and reliable due to its resemblance to classical cleft palate closure surgery as both the nasal side and the oral side closure provide more strength to the surgical closure site.

2- Contrary to free flaps, both layer flaps contain nutritional flap pedicles, as the orbicularis oris has its superior labial artery, and the cheek flap has its buccal arterial distal branches.

3- This technique does not cause any aesthetic or functional defects in the donor and recipient sites due to its proximity to the surgical area.

4- We think this new technique could be carried out under local anesthesia.

5- The richly vascular muscular flap was used for closure of the nasal side, and the less vascular cheek mucosal flap was used on the oral side to protect and strengthen this important nasal flap.

The only limitation of this technique is that we applied it to only one patient with dehiscence. Nevertheless, according to the literature that contains similar techniques working successfully, this new technique could be used in future problematic oronasal fistula closures, especially in anterior palate cases, to obtain more precise results [ 2 ].

## Conclusion

In cleft palate patients, recurrent surgeries are sometimes very challenging due to the usual fibrotic and less vascularity in the surgical site. In like these situations, cleft palate repair with vascular orbicularis oris muscle, plus buccal mucosal flap might be a good option for surgeons.

## Conflict of Interest

The authors declare that they have no conflict of interest.

## References

[1] Fang L, Yang M, Wang C, Ma T, Zhao Z, Yin N, et al ( 2014). A clinical study of various buccinator musculomucosal flaps for palatal fistulae closure after cleft palate surgery. J Craniofac Surg.

[2] Tiwari V, Sujata S ( 2006). Orbicularis oris musculomucosal flap for anterior palatal fistula. Indian J Plast Surg.

[3] Abdel-Aziz M, Abdel-Nasser W, El-Hoshy H, Hisham A, Khalifa B ( 2008). Closure of anterior post-palatoplasty fistula using superior lip myomucosal flap. Int J Pediatr Otorhinolaryngol.

[4] Hsu YT, Hao SP ( 2015). Repair of oronasal fistula with silicone button in patients with head and neck cancer. Eur Arch Otorhinolaryngol.

[5] Hardwicke JT, Landini G, Richard BM ( 2014). Fistula incidence after primary cleft palate repair: a systematic review of the literature. Plast Reconstr Surg.

[6] Honnebier MB, Johnson DS, Parsa AA, Dorian A, Parsa FD ( 2000). Closure of palatal fistula with a local mucoperiosteal flap lined with buccal mucosal graft. Cleft Palate Craniofac J.

